# Seasonal and Long‐Term Groundwater Unloading in the Central Valley Modifies Crustal Stress

**DOI:** 10.1029/2019JB018490

**Published:** 2020-01-20

**Authors:** G. Carlson, M. Shirzaei, S. Werth, G. Zhai, C. Ojha

**Affiliations:** ^1^ School of Earth and Space Exploration Arizona State University Tempe AZ USA; ^2^ School of Geographical Sciences and Urban Planning Arizona State University Tempe AZ USA; ^3^ Department of Earth and Planetary Science University of California Berkeley CA USA

**Keywords:** nontectonic stress, seasonal loading, groundwater, Central Valley, CA, Earthquake modulation

## Abstract

Changes in terrestrial water content cause elastic deformation of the Earth's crust. This deformation is thought to play a role in modulating crustal stress and seismicity in regions where large water storage fluctuations occur. Groundwater is an important component of total water storage change in California, helping to drive annual water storage fluctuations and loss during periods of drought. Here we use direct estimates of groundwater volume loss during the 2007–2010 drought in California's Central Valley obtained from high resolution Interferometric Synthetic Aperture Radar‐based vertical land motion data to investigate the effect of groundwater volume change on the evolution of the stress field. We show that GPS‐derived elastic load models may not capture the contribution of groundwater to terrestrial water loading, resulting in an underestimation of nontectonic crustal stress change. We find that groundwater unloading during the drought causes Coulomb stress change of up to 5.5 kPa and seasonal fluctuations of up to 2.6 kPa at seismogenic depth. We find that faults near the Valley show the largest stress change and the San Andreas fault experiences only ~40 Pa of Coulomb stress change over the course of a year from groundwater storage change. Annual Coulomb stress change peaks dominantly in the fall, when the groundwater level is low; however, some faults experience peak stress in the spring when groundwater levels are higher. Additionally, we find that periods of increased stress correlate with higher than average seismic moment release but are not correlated with an increase in the number of earthquakes. This indicates groundwater loading likely contributes to nontectonic loading of faults, especially near the Valley edge, but is not a dominant factor in modulation of seismicity in California because the amplitude of stress change declines rapidly with distance from the Valley. By carefully quantifying and spatially locating groundwater fluctuations, we will improve our understanding of what drives nontectonic stress and forces that modulate seismicity in California.

## Key Points

- Elastic vertical land motion due to groundwater volume loss is minute outside of the Central Valley
- Groundwater volume fluctuations in the Central Valley modulates crustal stress
- Seasonal stress changes due to water loading might modulate seismicity

## 1 Introduction

Fluctuations of water storage in a region reflect both climate and human activity and can be highly variable on interannual to interdecadal timescales (Rodell et al., 2018). Monitoring these storage changes is not only crucial for freshwater management but can also help us understand deformation and crustal stress patterns. Here we explore how to use the groundwater‐related poroelastic deformation signal, which shows subsidence in response to compaction at depth due to groundwater removal (Wang, 2000), to better understand the instantaneous regional elastic response of groundwater withdrawal and recharge in California's Central Valley. Measuring the solid Earth's elastic response to a change in surface mass has been explored in a number of hydrologic settings to evaluate terrestrial water storage (TWS) changes (Argus et al., 2014; Borsa et al., 2014; van Dam et al., 2001), surface water (Bevis et al., 2005; Wahr et al., 2013), snow water (Fu et al., 2012; Ouellette et al., 2013), ice (Sauber et al., 2000; Wahr et al., 2013), and groundwater (Amos et al., 2014; Holzer, 1979). Most often, GPS displacements have been used to invert for mass loss or gain using Green's functions based on the Preliminary Reference Earth Model (Dziewonski & Anderson, 1981). These estimates have been especially useful in assessing the impact of droughts on TWS, particularly in semiarid climates experiencing extreme water loss, like the western United States (Amos et al., 2014; Borsa et al., 2014).

In this study, we focus on the Central Valley aquifer‐system in California, which has recently undergone two severe, prolonged periods of drought (2007–2010 and 2012–2015). Both droughts have resulted in dramatic groundwater losses caused by intense pumping activities and reduced natural recharge (Famiglietti et al., 2011; Faunt et al., 2015; Ojha et al., 2018, 2019). Pumping in the Central Valley is not well documented; therefore, groundwater losses are difficult to quantify. Studies incorporating hydrogeology and hydrologic modeling have estimated groundwater withdrawal during dry years to be approximately 14.4 km^3^, which is almost 3 times higher than estimated in a typical wet year (Faunt, 2009). Here we focus on the elastic loading effects of seasonal groundwater fluctuations and the total volume loss from 2007 to 2010. During these years, estimates of the total groundwater loss range from 20.4 to 31.0 km^3^ (Famiglietti et al., 2011; Ojha et al., 2018; Scanlon et al., 2012).

In addition to the induced surface deformation, near‐surface hydrologic loading also has the potential to alter the local and regional stress field (e.g., Amos et al., 2014; Johnson et al., 2017a). Although these stress perturbations are relatively low amplitude, it has been suggested that elastic hydrologic loading encourages earthquake nucleation and may weakly modulate seismicity (Christiansen et al., 2007; Heki, 2003; Johnson et al., 2017a). In regions where elastic loading maintains a strong periodic signal, the same cyclic pattern is observed in seismic catalogs (Ader & Avouac, 2013; Christiansen et al., 2007; Heki, 2003), thus providing support for this hypothesis. The Central Valley exhibits strong seasonal loading signals and is bordered by networks of active faults, including the San Andreas Fault System. The San Andreas is dominated by right‐lateral strike‐slip motion associated with the boundary between the North American and Pacific plates. Amos et al. (2014) showed that seismicity rate peaks along the San Andreas in the dry fall season are associated with unclamping (reduced normal stress). Similarly, Johnson et al. (2017a) found that seasonal elastic loading due to TWS variations created periods of slip‐encouraging stress. Johnson et al. (2017a) also found that increased shear stress correlates with an increase in the number of earthquakes. Others have argued that these patterns are due at least in part to pore pressure diffusion (Hainzl et al., 2006) or seasonal thermoelastic strain (Ben‐Zion & Allam, 2013). While the statistical significance of seasonal variation in seismicity remains somewhat inconclusive (Christiansen et al., 2007; Heki, 2003; Johnson et al., 2017a), Coulomb stress, which represents the propensity for a fault to fail, has been shown to fluctuate with the wet and dry cycle on the order of ~1–2 kPa (Johnson et al., 2017a).

**Figure 1:**
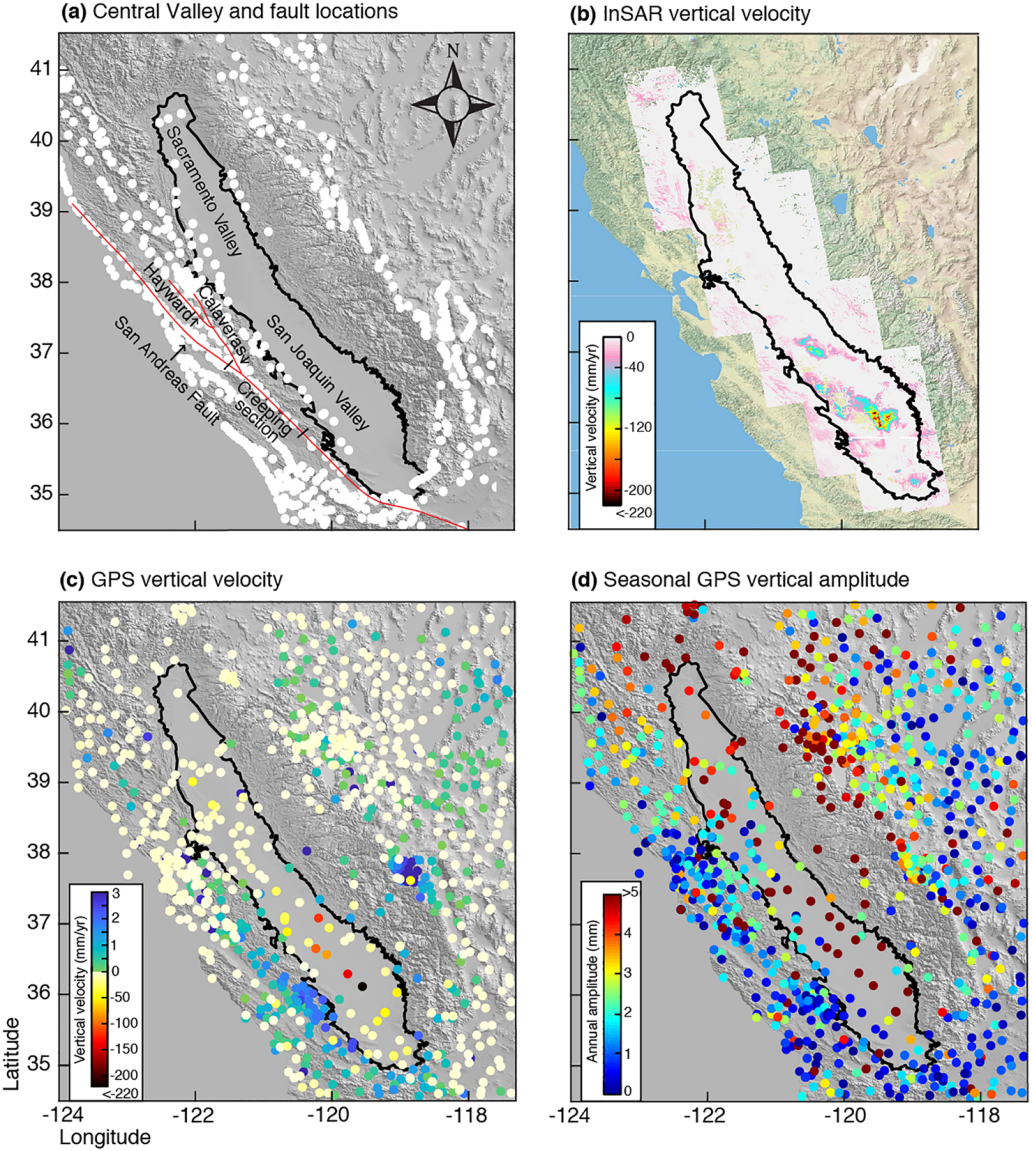
Study region and vertical land motion data. (a) Central Valley outlined in black with the northern Sacramento and southern San Joaquin basins identified. The San Andreas, Calaveras, and Hayward faults are outlined in red; other fault segments are shown by white dots (Field et al., 2013). (b) InSAR‐derived rate of vertical land motion using ALOS‐1 SAR images collected from December 2006 to January 2010 after Ojha et al. (2018). (c) GPS vertical land motion in and surrounding the Central Valley from observation periods of January 2007 to December 2009. (d) GPS seasonal amplitude of vertical displacements in and around the Central Valley. The GPS observations are provided by Nevada Geodetic Lab (http://geodesy.unr.edu/) and decomposed into annual and trend components.

Although Amos et al. (2014) reasoned that uplift of GPS stations surrounding the southern Central Valley is principally driven by groundwater loss, previous studies have not isolated the groundwater component of TWS change to calculate crustal stress change in California. This is likely because most studies use the elastic loading response of GPS stations, removing stations that exhibit a poroelastic response including those stations directly above aquifer systems, in the TWS inversion. Poroelastic deformation is caused by changing fluid pressure within an aquifer system with pumping and recharge. When groundwater is removed, pore pressure decreases, resulting in the closure of pore space and subsidence of the land surface. This is the opposite of the elastic response, which shows isostatic uplift as a response to groundwater loss, thus are not useful in inverse modelling the elastic displacements for the total water storage change. In this study, we use deformation from pore space compaction measured as vertical land motion (VLM) above the aquifer in order to better understand the groundwater contribution to the elastic land motion outside of the aquifer. We use estimates of groundwater volume loss and seasonal volume change in the Central Valley in California obtained by Ojha et al. (2018) during the 2007–2010 drought. Ojha et al. (2018) used a first‐order poroelastic calculation constrained by VLM measurements obtained from Interferometric Synthetic Aperture Radar (InSAR) shown in Figure 1b.

We forward model the elastic uplift response using these volume estimates and calculate the contribution of groundwater storage change to total crustal stress change during the drought and to the stressing amplitude. We then evaluate whether periods of increased stress change are correlated with periods of increased seismicity and seismic moment release. Groundwater loss during drought is primarily due to increases in pumping as less surface water is available for use (Faunt, 2009). Therefore, by isolating the groundwater component of TWS change during the 2007–2010 drought, we will better understand how anthropogenic sources of water loss contributes to crustal stressing rates and consider whether it contributes to seismogenesis.

## 2 Materials and Methods

### 2.1 VLM and Groundwater Volume Loss

VLM can be measured using a number of geodetic and remote sensing techniques. GPS stations have been shown to resolve elastic solid earth deformation due to mass redistributions (Argus et al., 2014; Bevis et al., 2005; Borsa et al., 2014; Fu et al., 2012; Ouellette et al., 2013; Sauber et al., 2000; van Dam et al., 2001; Wahr et al., 2013). However, it has also been observed that GPS stations are more sensitive to loads in the direct vicinity of stations than to loads at further distances, making large‐scale loading calculations more challenging (Bevis et al., 2005; Khan et al., 2010). Additionally, inversions using GPS uplift signals to resolve loads other than single‐point masses cannot be uniquely determined, especially if GPS station density is low or the load is spread over a broad region (Wahr et al., 2013). Apart from GPS inversions, load changes due to the redistribution of water can be estimated using data from the GRACE twin satellites, which estimate small disturbances to the gravity field on a monthly time‐scale. The primary disadvantage with these load change measurements is their coarse spatial resolution (~200–300 km). Spaceborne InSAR has also been proven useful in measuring compaction due to the removal of fluid (Galloway et al., 1998; Miller et al., 2017; Ojha et al., 2019) and, thus, can be used to estimate groundwater volume loss (Ojha et al., 2018). Here we use published groundwater volume loss estimates from VLM (Ojha et al., 2018) found using a combination of InSAR line of sight (LOS) displacements and horizontal displacements from Plate Boundary Observatory network GPS stations to evaluate the effect groundwater loss has on vertical elastic deformation outside of the Valley and changes to the magnitude of stress along faults in California.

InSAR LOS displacements were measured by Ojha et al. (2018) using an advanced multitemporal Wavelet‐Based InSAR algorithm (Shirzaei, 2013; Shirzaei & Bürgmann, 2012). 420 L‐band SAR images were acquired on ascending tracks from the ALOS‐1 satellite during the drought period (26 December 2006 to 1 January 2010). From these SAR images, more than 1,600 interferograms were generated. Elite (i.e., less noisy) pixels were identified, and wavelet‐based filters were used to correct topographically correlated atmospheric phase delay (Shirzaei & Bürgmann, 2012) and spatially uncorrelated DEM error (Shirzaei, 2013; Shirzaei & Bürgmann, 2012). Using a reweighted least squares method, the interferometric data set was inverted to solve for the time series of LOS displacement at each pixel. Lastly, a wavelet‐based high‐pass filter was applied to reduce the effect of temporally uncorrelated residual atmospheric errors. Details on combining InSAR and GPS measurements to estimate the vertical land motion are previously published (Ojha et al., 2018; Shirzaei & Bürgmann, 2018). Ojha et al. (2018) also apply a least squares spectral analysis to extract seasonal components of the deformation field for each pixel. Ojha et al. (2018) show that InSAR VLM agrees well with GPS observations with an overall standard deviation of the difference of ~1 cm/year. Discrepancies between InSAR and GPS vertical velocity are larger in some parts of the study area (e.g., over the Coast Ranges), which could be a result of residual horizontal displacement and topography correlated atmospheric delay.

Seasonal groundwater volume change and total groundwater volume loss are determined through a first‐order poroelastic calculation constrained using the InSAR‐derived vertical displacements (Ojha et al., 2018). Using the 1‐D relationship between vertical strain, *dϵ*_*v*_, and effective stress, *dσ*′, change in volume, *dv*, is calculated for subsiding pixels with an area, *A*, using the equation:

(1) $$\mathrm{dv}=\mathrm{Adh}=-A\frac{d\sigma^{\prime }}{\alpha_{\beta }\rho_{w}g}=\frac{\mathrm{Ad}\epsilon_{v}E}{\alpha_{\beta }\rho_{w}g}$$

where *E* is the bulk modulus equal to 200 MPa in the Sacramento Valley (Figure 1a) and 300 MPa in the San Joaquin Valley (Figure 1a) (Ojha et al., 2018), *α*_*β*_ is the Biot‐Wills coefficient describing the ratio of pore fluid pressure to confining stress, here set equal to 0.9, *ρ*_*w*_ is the density of water, and *g* is the gravitational acceleration.

**Figure 2:**
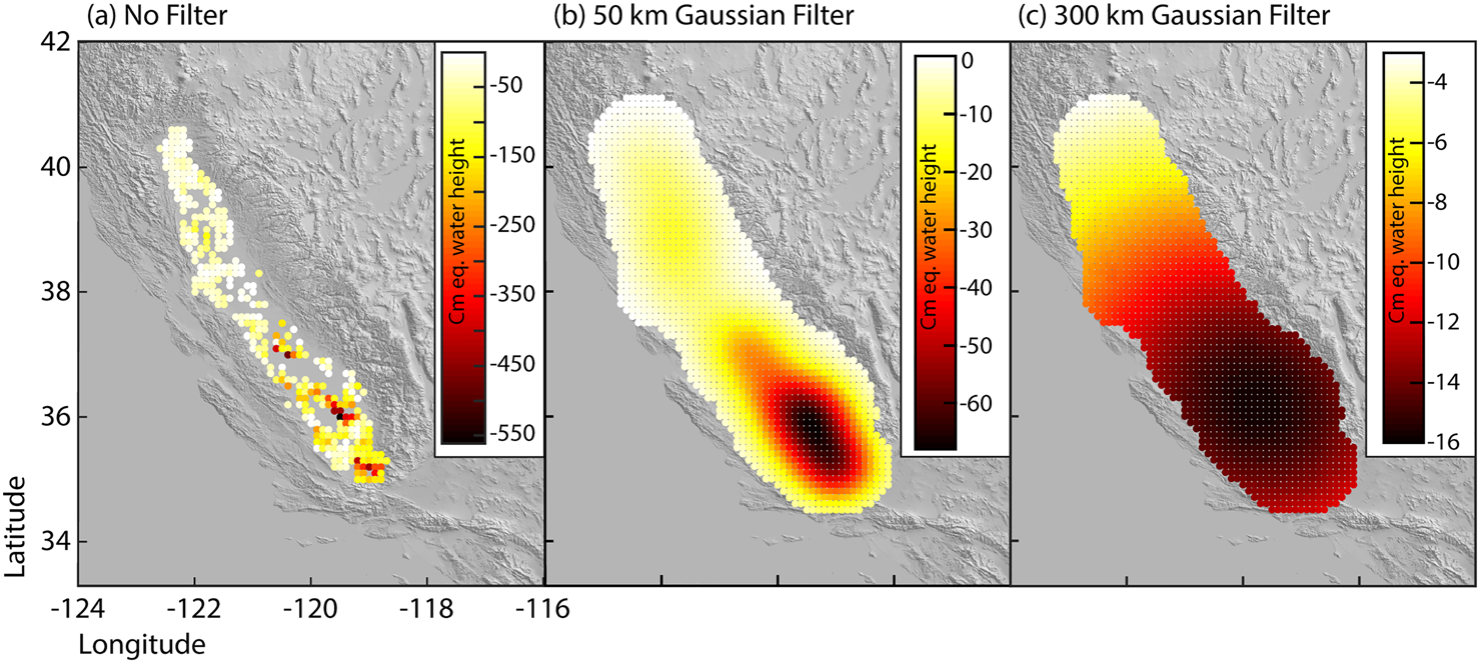
Total centimeter equivalent (cm‐eq.) water height of groundwater lost during the 2007–2010 drought. (a) Cm‐eq. water height derived from VLM data (Ojha et al., 2018) aggregated to 0.1° resolution. Cm‐eq. water height from (a) is smoothed using (b) 50‐ and (c) 300‐km Gaussian smoothing filters. Note the color scales are different on each panel because the filters cause the largest mass loss to be distributed over a broader region.

Ojha et al. (2018) used the vertical land motion data to estimate groundwater volume at a resolution of 0.002° grid cell spacing (~400 m^2^). We aggregate pixel values to achieve a spatial resolution of 0.1° (~100 km^2^) and convert the volume change to centimeter equivalent (cm‐eq.) water height (Figure 2a). In order to evaluate the effect that spatial resolution has on the results, we convert the 0.1° resolution cm‐eq. water height data into a spherical harmonic (SH) representation. Then we downweight, that is, filter, the corresponding SH coefficients with Gaussian filters of 50‐ (Figure 2b) and 300‐km radius (Figure 2c) using the SH‐bundle software from the Institute of Geodesy at the University of Stuttgart (https://www.gis.uni‐stuttgart.de/en/research/downloads/shbundle/). In the spatial domain, the 300‐km filter has a similar resolution as GRACE satellite data. Therefore, the pattern of crustal stress change using this smoothing filter would be similar to results that we would obtain using only the groundwater component derived from GRACE observations. The filtered SH functions are converted back to a gridded data set of volume changes in units of cm‐eq water height. After the smoothed SH functions are converted back into a spatial grid, many points outside of the Valley show a nonzero equivalent water height due to the damping effect of the filter (visible in Figure 2c); however, to further limit the number of observation points, we remove pixels outside of a 0.5° buffer surrounding the Central Valley.

**Figure 3:**
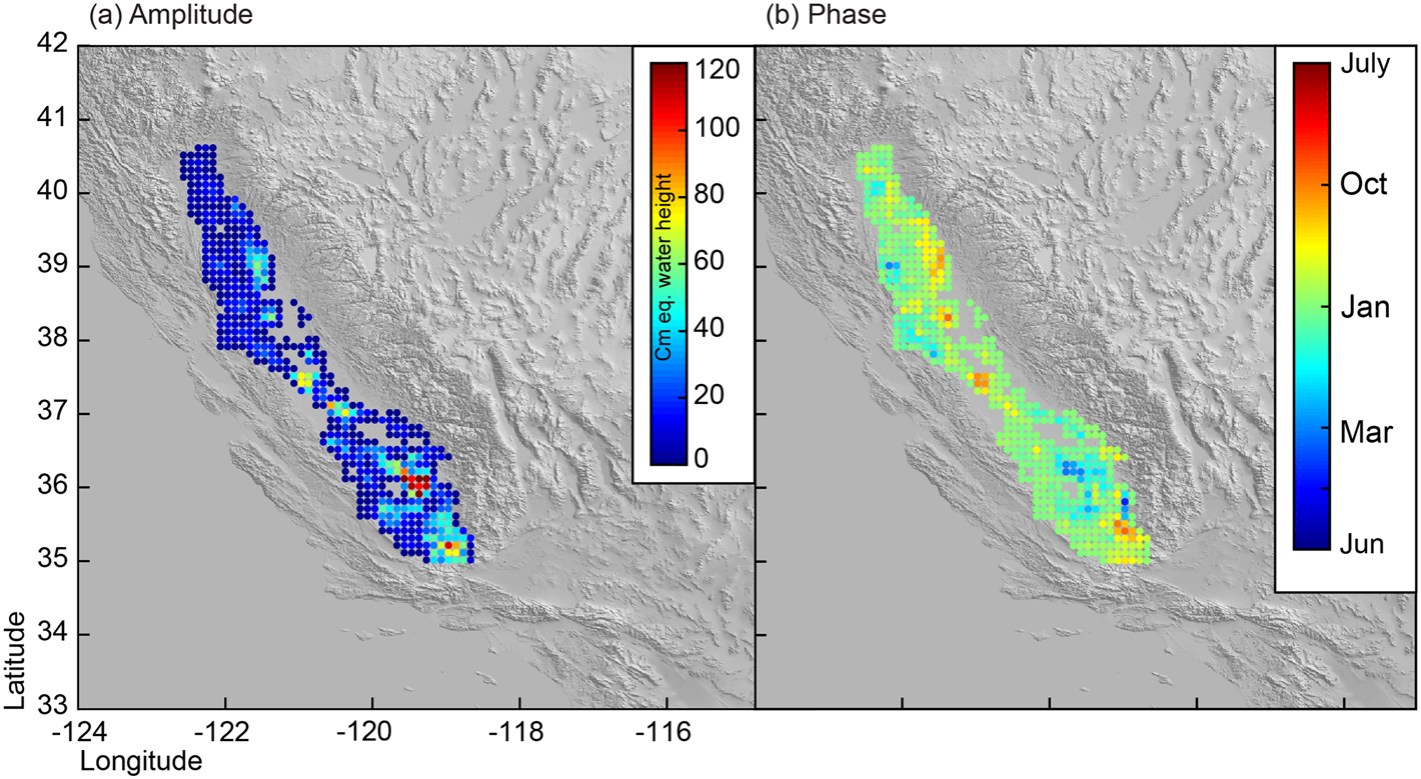
(a) Centimeter equivalent water height seasonal amplitude aggregated to 0.1° resolution and (b) phase of seasonal amplitude averaged to 0.1° resolution. Both data sets were estimated from VLM observations provided by Ojha et al. (2018).

We also use seasonal volume change amplitude and phase estimates from Ojha et al. (2018). Once again, seasonal amplitude observations are aggregated to 0.1° resolution, and the phase estimates are averaged to 0.1° resolution. Comparing Figures 2a and 3a, we can see that seasonal volume change amplitudes are more consistent across the entire Valley, although larger seasonal amplitudes occur in the southern part of the Valley where aquifers have larger elastic skeletal storage coefficients (Faunt, 2009; Ojha et al., 2018). This observation is also consistent with water use (Hanak et al., 2017). The smoothed solutions (Figures 2b and 2c) show groundwater loss centered in the southern Central Valley, but the loss is more distributed.

Throughout most of the Valley, peak groundwater recharge occurs following the wet winter, due to reduced pumping stimulated by an abundance of surface water (Faunt, 2009), indicated by the green‐colored phase observations (Figure 3b). Unlike the rest of the Valley, pockets of the San Joaquin Valley in the south, where groundwater recharge and discharge is controlled by agricultural activity, show peak recharge during the irrigation season in the spring, indicated by blue colors. During this time, surface water deliveries are made available to farmers in the southern Central Valley so that farmers require less groundwater (Hanak et al., 2017). Excess irrigation water can also flow down through perforated groundwater well casings, which increase the vertical hydraulic conductivity between the shallow unconfined and deeper confined and semi‐confined aquifers and aid in aquifer recharge during this time (Faunt, 2009). It is also important to note that the largest seasonal amplitudes occur where we see peak recharge in the spring versus in the winter, showing that the largest seasonal recharge and discharge rates are dominantly controlled by agricultural activity (Ojha et al., 2019; Shirzaei et al., 2019).

### 2.2 Forward Modeled Vertical Displacement Due to Groundwater Unloading

By convolving Green's functions for a gravitating, spherical, layered Earth based on load‐Love numbers from the Preliminary Reference Earth Model (Dziewonski & Anderson, 1981) with surface load, we generate theoretical displacements due to the removal of a mass. The unit mass, 𝑀, is uniformly distributed over the area of each disk, 𝐴_𝑠_, with radius, α = 6 km, as a function of the angular distance, 𝜃, from the center of the load (Farrell, 1972):

(2) $$\frac{M}{A_{s}}=\rho_{w}*\frac{V}{\pi \alpha^{2}}=\sum_{n=0}^{\infty }\Gamma_{n}P_{n}\left(\cos\;\theta \right)$$

where the *P*_*n*_ are Legendre polynomials, *ρ*_*w*_ is the density of water, and

(3) $$\Gamma_{n}=\left\{\begin{array}{c} \left[P_{n-1}\left(\cos\alpha \right)-P_{n+1}\left(\cos\alpha \right)\right]/\left[4\pi r_{E}^{2}\left(1-\cos\alpha \right)\right],\;n>0\\ \frac{1}{4}\pi r_{E}^{2},\;n=0 \end{array}\right.$$

where *r*_*E*_ is the radius of the Earth, equal to 6,378.14 km. Each disk is approximately the size of 0.1° with a height equal to the rate of centimeter‐equivalent water height lost. The addition or removal of the mass causes a perturbation of the gravitational field. The transformed surface potential is (Farrell, 1972) as follows:

(4) $$\Phi_{n}=\frac{4\mathrm{\pi G}r_{E}}{2n+1}\Gamma_{n}$$

where *G* is Newton's gravitational constant. This perturbation results in displacements in the vertical, *S*_*up*_, and horizontal, *S*_*away*_, and are given by (Farrell, 1972)

(5) $$S_{\mathrm{up}}=\sum_{n=0}^{\infty }\Phi_{n}\frac{h_{n}}{g}P_{n}\cos\;\theta \;$$

(6) $$S_{\text{away}}=\sum_{n=0}^{\infty }\Phi_{n}\frac{l_{n}}{g}\frac{{\partial P}_{n}\cos\;\theta \;}{\partial \cos\;\theta \;}$$

where *g* is gravitational acceleration and *h*_*n*_ and *l*_*n*_ are the load‐Love numbers.

Mass loss across the Valley is spatially variable; therefore, we need to understand the combined loading effect from all of our observation points. Given the linearity of equations, vertical displacement caused by multiple masses is superimposed to find the total vertical displacement at locations surrounding the Valley. In this study, we only consider the vertical displacement. As pointed out by Argus et al. (2017) and Chanard et al. (2014), the choice of Earth model is important. We use a gravitating, spherical, layered Earth model, not a nongravitating elastic half‐space model, as in Amos et al. (2014). Thus, we do not expect our forward‐modeled uplift, particularly at the center of the load, to be comparable to Amos et al. (2014).

### 2.3 Calculating Stress Change

Stress is calculated at seismogenic depth (8 km) along faults using the UCERF3 fault model (Field et al., 2013) and at the locations of a declustered set of earthquake focal mechanism solutions for earthquakes occurring between January 2006 and December 2014 and with M≥ 2.0 (Johnson et al., 2017a). Focal mechanism fault planes are differentiated from the auxiliary nodal plane by Johnson et al. (2017a) based on orientation: for strike‐slip mechanisms, the fault plane closest to the strike of the San Andreas fault (~325°) is chosen. For dip‐slip mechanisms, the fault plane closest to Andersonian geometry is chosen (dips of 30° for reverse faults and 60° for normal faults).

To find the six‐component strain tensor at these locations and at a depth of 8 km, which is the approximate average depth of earthquakes in the catalogue (Johnson et al., 2017a), we consider the total volume loss during the drought (Figure 2) and seasonal volume change (Figure 3) calculated at each of our observation points and convert this to a force at the surface. We then apply a modified version of STATIC1D, used by Johnson et al. (2017a, 2017b), for spherical harmonic degree up to 3500 (Pollitz et al., 2013; Pollitz, 1996; Courtesy F. Pollitz). We convert the strain tensor [*ϵ*_*xx*_, *ϵ*_*yy*_, *ϵ*_*zz*_, *γ*_*xy*_, *γ*_*xz*_, *γ*_*yz*_]^*T*^ to the stress tensor [*σ*_*xx*_, *σ*_*yy*_, *σ*_*zz*_, *τ*_*xy*_, *τ*_*xz*_, *τ*_*yz*_]^*T*^ through a linear transformation of Hooke's Law:

(7) $$\left[\begin{array}{c} \begin{array}{c} \begin{array}{c} \begin{array}{c} \begin{array}{c} \sigma_{\mathrm{xx}}\\ \sigma_{\mathrm{yy}} \end{array}\\ \sigma_{\mathrm{zz}} \end{array}\\ \sigma_{\mathrm{xy}} \end{array}\\ \sigma_{\mathrm{xz}} \end{array}\\ \sigma_{\mathrm{yz}} \end{array}\right]=\left[\begin{array}{ccc} \begin{array}{cc} \begin{array}{c} \begin{array}{c} \begin{array}{c} M\left(1-v\right)\\ Mv \end{array}\\ Mv \end{array}\\ 0\\ 0\\ 0 \end{array} & \begin{array}{c} \begin{array}{c} \begin{array}{c} Mv\\ M\left(1-v\right) \end{array}\\ Mv \end{array}\\ 0\\ 0\\ 0 \end{array} \end{array} & \begin{array}{c} \begin{array}{c} \begin{array}{c} Mv\\ Mv \end{array}\\ M\left(1-v\right) \end{array}\\ 0\\ 0\\ 0 \end{array} & \begin{array}{c} \begin{array}{c} 0\\ 0\\ 0 \end{array}\\ G\\ 0\\ 0 \end{array} \end{array}\;\begin{array}{c} \begin{array}{c} 0\\ 0\\ 0 \end{array}\\ 0\\ G\\ 0 \end{array}\;\begin{array}{c} \begin{array}{c} 0\\ 0\\ 0 \end{array}\\ 0\\ 0\\ G \end{array}\right]\left[\begin{array}{c} \begin{array}{c} \in_{\mathrm{xx}}\\ \in_{\mathrm{yy}}\\ \in_{\mathrm{zz}} \end{array}\\ \gamma_{\mathrm{xy}}\\ \gamma_{\mathrm{xz}}\\ \gamma_{\mathrm{yz}} \end{array}\right]$$

where *ν* is Poisson's ratio, equal to 0.25, *Ε* is Young's modulus, equal to 50 × 10^9^ Pa, $G=\frac{Ε}{2\left(1+\nu \right)}$ is the shear modulus, and $Μ=\frac{Ε}{\left(1-2\nu \right)\left(1+\nu \right)}$ is an effective elastic modulus.

We project stress tensors in fault parallel (*σ*_*s*_) and perpendicular (*σ*_*n*_) directions. Assuming a friction coefficient of *μ* = 0.4, the Coulomb failure stress change is as follows:

(8) $$∆\mathrm{CFS}=\sigma_{s}+{\mu \sigma }_{n}.$$

The *∆CFS* is also calculated for each month throughout the year using seasonal volume change amplitudes and phase estimates. We find the monthly load force (*f*^*t*^_*i*_) using the force of the seasonal amplitude (*f*^*A*^_*i*_) and phase (*φ*_*i*_) for each observation point, *i*:

(9) $${f^{t}}_{i}={f^{A}}_{i}\times \text{sin}\;\left(2\mathrm{\pi t}+\varphi_{i}\right),t=0,\frac{1}{12},\frac{2}{12},\ldots ,\frac{11}{12}\;$$

### 2.4 Correlating Stress Change With Seismicity

Periodic stress perturbations have been suggested by observations of both seismicity in natural fault systems (e.g., Christiansen et al., 2007; Bettinelli et al., 2008), laboratory experiments (e.g., Beeler & Lockner, 2003; Lockner & Beeler, 1999), and numerical simulations (Lockner & Beeler, 1999) to exert control on earthquake nucleation. Many studies have focused on the number of events (e.g., Christiansen et al., 2007; Bettinelli et al., 2008). The underlying theory has been explained by periodic loading simulations, which suggest that if the stressing amplitude is of the same magnitude and the period is sufficiently larger than the nucleation time, then the timing of earthquakes might be controlled by these periodic stress perturbations (Ader et al., 2014). These simulations have also shown that in some cases, stress perturbations can change the balance between small and large earthquakes, thus changing the seismic moment release while having little or no effect on the total number of events (Ader et al., 2014). This idea was also considered by Kreemer and Zaliapin (2018), who observed that mainshock intensity was correlated with Coulomb stress perturbations, but not always correlated with an increase in the number of events. We test for both an increase in the number of events and an increase in the total seismic moment release.

To test whether we can observe periodic increases in seismicity in our focal mechanism catalog that matches periodic stress increases due to groundwater volume change, we compare the total number of earthquakes in a synthetic seismic catalog to the total number of earthquakes in our observed catalog. We generate our synthetic catalog by considering a physics‐based seismicity rate model that solves for the change in seismicity rate over the change in time ( $\frac{\mathrm{dR}}{\mathrm{dt}}$), assuming a constant tectonic stressing rate (Dieterich, 1994):

(10) $$\frac{\mathrm{dR}}{\mathrm{dt}}=\frac{R{\dot{\tau }}_{0}}{A\bar{\sigma }}\left(\frac{\dot{\tau }}{{\dot{\tau }}_{0}}-R\right)$$

where *R* is the seismicity rate relative to the background rate, ${\dot{\tau }}_{0}$ is the background stressing rate, *A* is a rate‐and‐state parameter from laboratory experiments, 𝜎̅ is the effective normal stress and $\dot{\tau }$ is the change in Coulomb stress over a change in time. In our case, we keep *R* = 1, resulting in a constant seismicity rate over time. If *R* is greater than 1 or less than 1, this would indicate earthquake triggering or arresting, respectively, due to some temporal change in stress. Based on the theory behind this seismicity rate model, we simulate an earthquake‐magnitude time distribution using a seismicity rate model that incorporates the physics of earthquake nucleation with a well‐known nonhomogenous Poissonian process that captures the randomness of the earthquake distributions (Segall & Lu, 2015). The earthquake count per unit area, *x,* per unit time, *t*, per unit magnitude, *M*, is given by (Zhai et al., 2019; Zhai & Shirzaei, 2018)

(11) $$R\left(x,t,M\right)=\frac{\ln\left(10\right)\mathrm{kb}10^{-\mathrm{bM}}}{S_{0}}R\left(x,t\right)$$

where *k* is the annual rate given by 10^*a*^ for a region with the size, *S*_0_, and *a* and *b* are two parameters associated with the Gutenberg‐Richter frequency‐magnitude distribution. We use *k* = 3.33 × 10^4^ that is extrapolated from figures in Tormann et al. (2014) and Felzer (2013) and *b*, which characterizes the earthquake size distribution, is set equal to 1 after Felzer, (2013). The total number of earthquakes per unit time per unit magnitude is given by integrating over the entire area, *S* (Zhai et al., 2019; Zhai & Shirzaei, 2018):

(12) $$R\left(t,M\right)=\int_{S}R\left(x,t,M\right)\mathrm{dx}=\frac{\ln\left(10\right)\mathrm{kb}10^{-\mathrm{bM}}}{S_{0}}\int_{S}R\left(x,t\right)\mathrm{dx}.$$

Next, we simulate the magnitude‐time distribution governed by the function *R*(*t*,*M*) by discretizing time, *t*, into *N*_*t*_ evenly spaced time samples $\left[t_{1},\;t_{2},\ldots ,\;t_{i},\ldots ,\;t_{N_{t}}\right]$ with time interval length of *∆t* = *t*_*i*+1_ − *t*_*i*_ and define the minimum magnitude, *M*_*min*_, and maximum magnitude, *M*_*max*_, to mimic a probability distribution. We set *M*_*min*_ to 0 and *M*_*max*_ to 10 and calculate the total number of earthquakes (Zhai et al., 2019; Zhai & Shirzaei, 2018):

(13) $$N\left(t_{i}\right)=\int_{M_{\min}}^{M_{\max}}\int_{t_{i}}^{t_{i+1}}R\left(t,M\right)\text{dtdM}.$$

We define a cumulative probability distribution as a function of earthquake magnitude (Zhai et al., 2019; Zhai & Shirzaei, 2018)

(14) $$P\left(M;t_{i}\right)=1-\frac{\int_{M}^{M_{\max}}\int_{t_{i}}^{t_{i+1}}R\left(t,M\right)\text{dtdM}}{\int_{M_{\min}}^{M_{\max}}\int_{t_{i}}^{t_{i+1}}R\left(t,M\right)\text{dtdM}}$$

and randomly sample this distribution over the time interval [*t*_*i*_ *t*_*i*+1_] for *N*(*t*_*i*_) earthquakes and iterate over the entire time period to determine the magnitude‐time distribution.

Our synthetic catalogue is inherently declustered because the basis for our seismicity rate model only considers independent events (Dieterich, 1994). After ensuring our synthetic catalog contains a similar magnitude‐frequency relationship with our observed catalog, we remove all events with magnitudes less than 2.0. Despite the uncertainties associated with the *a* and *b* parameters, Figure S3 in the supporting information shows that the synthetic and real catalogues have similar magnitude‐frequency distributions. Thus, we believe the parameters chosen here are appropriate.

We solve for the percent excess seismicity (*P*_*excess*_) in each month using

(15) $$P_{\text{excess}}=\frac{N_{\text{Tsynth}}}{N_{\text{Tobs}}}*100*\left(N_{\mathrm{obs}}-N_{\text{synth}}\right)/N_{\text{synth}}$$

where *N*_*obs*_ and *N*_*synth*_ are the number of earthquakes in a given month or year in our observed and synthetic catalogs, respectively, and $\frac{N_{\text{Tsynth}}}{N_{\text{Tobs}}}$ is a normalization factor used to account for differences in the total number of earthquakes in our observed, *N*_*Tobs*_, and synthetic, *N*_*Tsynth*_, catalogs.

We generate 500 catalogs by shuffling the original catalog and replacing 5% with new values from the probability distribution. With each iteration, we calculate the percent excess and correlation coefficient, removing outliers (significance of <0.95) to establish a robust estimate. We use the average percent excess seismicity in each 1‐month period and correlate this with the mean stress change for values greater than 0.05 kPa or less than −0.05 kPa for earthquakes that occur in each 1‐month bin.

To look at the effect of periodic stress on the seismic moment, we correlate the total seismic moment release with mean stress in each monthly bin. The total seismic moment release is solved by summing the seismic moment release of all earthquakes in each respective time interval using the magnitude, *M*, of each event (Hanks & Kanamori, 1979; Kanamori, 1977)

(16a) $$M_{o}=10^{\left(\frac{3}{2}*M\right)+16.1.}$$

This calculation is for magnitudes with type M_w_. Earthquakes less than magnitude 3–3.5 are typically type either *M*_*L*_ or *M*_*d*_. For these earthquakes (*M* < 3), we use 16b after (Bakun, 1984).

(16b) $$M_{o}=10^{\left(1.2*M\right)+17.0}$$

M_L_ and M_d_ are reported to agree for magnitudes between 1.5 and 3.25 (Bakun, 1984). We then solve for the percent excess seismic moment (*P*_*excess*_) in each month using

(17) $$P_{\text{excess}}=\frac{{\mathrm{TM}}_{\text{synth}}}{{\mathrm{TM}}_{\mathrm{obs}}}*100*\left(M_{o_{\mathrm{obs}}}-M_{o_{\text{synth}}}\right)/M_{o_{\text{synth}}}$$

where $M_{o_{\mathrm{obs}}}$ and $M_{o_{\text{synth}}}$ are the total seismic moment released in a given month using our observed and synthetic catalogs, respectively, and $\frac{{\mathrm{TM}}_{\text{synth}}}{{\mathrm{TM}}_{\mathrm{obs}}}$ is a normalization factor used to account for differences in the total moment released for all earthquakes in our observed, *TM*_*obs*_, and synthetic, *TM*_*synth*_, catalogs. We use the same procedure for excess seismic moment release as for excess seismicity.

## 3 Results

### 3.1 Comparison of Forward‐Modeled and Measured GPS Vertical Displacements

**Figure 4:**
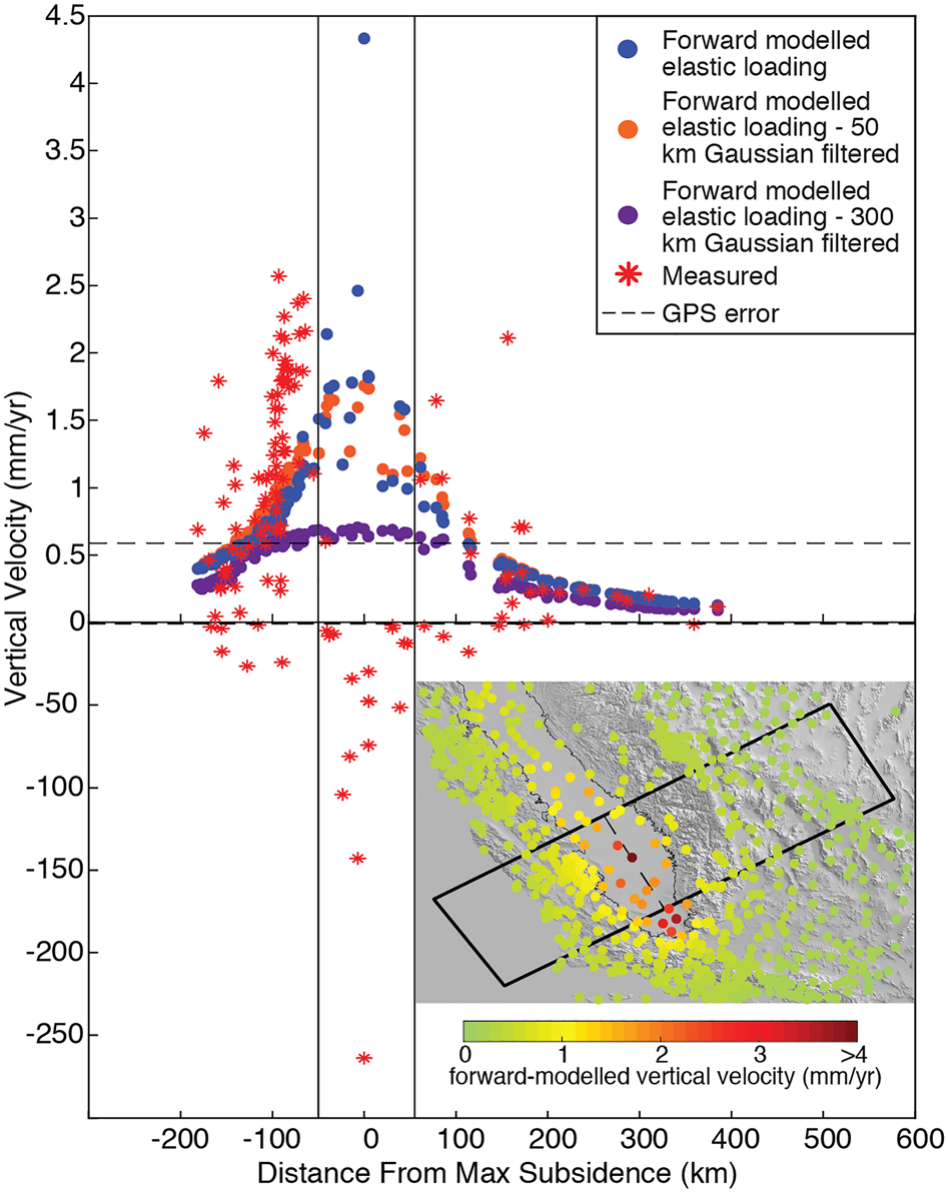
Measured GPS vertical displacement rate compared to the predicted vertical displacement rate from the elastic response to groundwater loss in the Central Valley. Vertical lines are the approximate edges of the Valley. GPS stations are taken from a 200‐km width centered on the station showing greatest subsidence, shown in the lower right inset map. Dashed black line shows mean GPS vertical velocity error of the stations included. Blue dots show forward‐modeled vertical velocity of elastic groundwater unloading. Red stars show measured GPS vertical velocity from Nevada Geodetic Laboratory for each station. Purple dots show predicted vertical velocity using the 300 km (orange), meant to show similar resolution to what is achievable using GRACE, and Orange dots show predicted vertical velocity using the 50‐km Gaussian filtered data set, an intermediate level of smoothing between our unfiltered and 300‐km Gaussian filtered data set. All rates are given in mm/year. Note scales on vertical axis are different above and below “0.” Inset map shows locations of GPS stations used (colored to forward‐modeled vertical velocity. All measured GPS observations span 1 January 2007 to 31 December 2009. The axis of the Valley is shown by the grey line in the inset map. All distance is calculated from the dotted line. Black box outlines the region of interest.

We forward model vertical displacements at the location of GPS stations within and surrounding the Valley. GPS time series are from the Nevada Geodetic Laboratory (Blewitt et al., 2018) and are separated into annual (Figure 1d) and trend (Figure 1c) components using a least squares additive decomposition method. We measure the distance between GPS station locations and the center of the Valley by defining a line that crosses the point of maximum subsidence (latitude 35.24, longitude −119.72) and is oriented parallel to the Valley then calculate the distance from that line to each GPS station. We compare forward‐modeled vertical velocity due to elastic loading at the location of GPS stations and compare them with measured vertical GPS velocities within a 200‐km‐wide band shown by the black box in the inset map (Figure 4). We choose stations with complete time series from 1 January 2007 to 31 December 2009 (a total of 193 stations). GPS vertical velocity error in the form of standard deviation is provided for each station by the Nevada Geodetic Laboratory. The mean standard deviation is shown by the black dashed line in Figure 4. Locations of each station are shown in the inset in Figure 4, colored to the forward‐modeled elastic uplift.

Most GPS stations located in the Valley (indicated by the two vertical lines) show a large, poroelastic response to long‐term groundwater loss, causing subsidence rates of up to −263.6 mm/year. This subsidence rate dwarfs the modeled elastic uplift rate of only +4.3 mm/year, which peaks in the same area where we find the largest measured subsidence rates. At the edge of the Valley, calculated elastic vertical displacement due to groundwater unloading drops to 1.5 mm/year or less. Long‐term uplift rates due to elastic groundwater unloading at stations further than ~110 km from the center of greatest subsidence are below the estimated trend error. In the Sierra Nevadas, predicted elastic uplift during this drought is less than 1 mm/year while in the Coast Ranges, the elastic uplift rate is ~1–1.2 mm/year.

The orange and purple points show the forward‐modeled vertical velocity curves using the 50‐ and 300‐km Gaussian smoothing filters. If we approximate the groundwater volume loss as removing a load in the shape of a disk, the two filters show the effect of changing the radius and height of the disk. The 300‐km Gaussian filter has approximately the radius of a disk that can be resolved using the GRACE satellites, while the 50‐km Gaussian filtered data is an intermediate disk radius. We can see that the vertical velocity curve becomes overall smaller and broader as the disk radius becomes larger, and the disk height becomes shorter. The differences in these three models show that elastic load models are highly sensitive to the shape of the load added or removed. Higher spatial resolution measurements will permit a more accurate representation of the shape and amplitude of the load. Some GPS stations within ~100 km of the edge of the Valley match the expected elastic uplift response to groundwater loss for both the unfiltered and the 50‐km Gaussian smoothed model. We find that in all models, but especially in the unfiltered model, the elastic uplift signal dissipates rapidly past the edge of the Valley and subsidence due to poroelastic compaction in the Valley overprints most of the elastic uplift signal. This leaves only a few stations that can detect any elastic uplift related to groundwater changes. Those that can, do not detect the peak uplift signal. Thus, loading studies based on the GPS observations outside the Valley may not detect the entire groundwater component of TWS change. Additionally, studies using GPS displacements that wish to separate the nontectonic elastic loading signal and the tectonic signal must choose the correct loading model to isolate the signal of interest accurately. Thus, this result has significant consequences not only for accurate inverse modeling of TWS changes but also for calculations of nontectonic and tectonic loading on nearby faults.

### 3.2 Stress Field Perturbation Due to Long‐Term Groundwater Unloading

**Figure 5:**
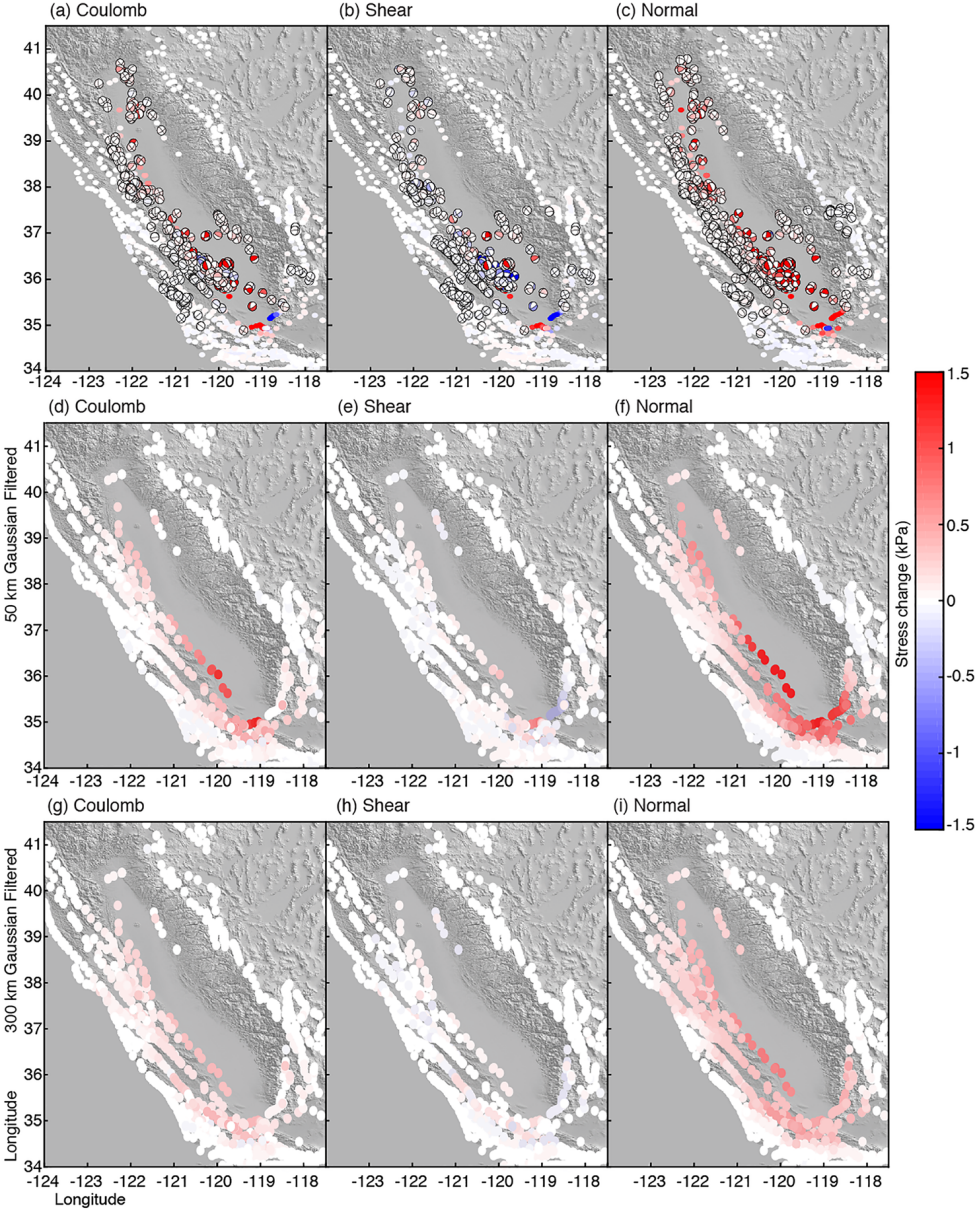
Stress field perturbation along faults in California from total groundwater loss during the 2007–2010 drought. Columns (right left) show Coulomb, shear, and normal stress change using (a–c) unfiltered data (row 1), (d–f) data smoothed using a 50‐km Gaussian smoothing filter (row 2), and (g–i) data smoothed using a 300‐km Gaussian smoothing filter (row 3).

We calculate the change in the stress field caused by groundwater loss during the drought on focal mechanism fault planes at a depth of 8 km and on fault planes using the UCERF3 fault model. Figure 5 shows the Coulomb, normal, and shear stress change using the unfiltered volume loss data (5a), the 50‐km Gaussian filtered (5b), and the 300‐km Gaussian filtered (5c) models. For normal stress, we follow the convention that tension is positive, and compression is negative.

Comparing these three data sets, we can see that spatial resolution impacts the stress change rate, particularly the shear stress change rate. The unfiltered model shows primarily larger negative shear stress change along faults in California, while the smoothed models show very small and dominantly positive shear stress change rates. For all models, the Coulomb and normal stress change are dominantly positive. The 50‐km Gaussian smoothed model has the largest normal stress change along the San Andreas fault. The 300‐km Gaussian filtered model shows a more uniform and slightly lower stress change than the other two models. This is likely because the width of the filter is larger than the valley itself, so the signal is smeared outside of the study area.

Only ~70% of the focal mechanisms experience stress greater than 10 Pa, so many faults in California feel no effect from groundwater loss within Central Valley during this drought. Some faults experience stress change as high as 5.5 kPa or as low as −1.1 kPa. These faults are very close to the Valley edge. We have no focal mechanisms on the south end of the Valley; however, using the UCERF model, we can see that these faults experience some of the largest stress change of ~1.5 kPa. Some portions of San Andreas fault experiences ~0.1–0.3 kPa of stress change.

The 50‐km Gaussian filtered data shows ~0.8–1.3 kPa of stress change along the faults on the southwest and south sides of the Valley. The San Andreas fault experiences ~0.2–0.4 kPa of stress change, which is larger than using the unfiltered data. We find Coulomb stress change of no more than 0.4 kPa or no less than −0.2 kPa when we use the 300‐km Gaussian filtered data. Thus, smoothing the groundwater load with this filter significantly underestimates the stress change.

### 3.3 Stress Field Perturbation Due to Seasonal Groundwater Unloading

We calculate stress change for seasonal loads using the phase and amplitude estimates of volume loss. We do not account for interannual variation in seasonal amplitude, because they are not captured by sparse acquisitions of the ALOS‐1 satellite used by Ojha et al. (2018). During times of drought, the amplitude of seasonal storage change is damped due to low recharge and a faster rate of loss. Therefore, Coulomb stress change estimates based on seasonal amplitudes during periods of drought are likely underestimated as compared to seasonal amplitudes during a typical year.

**Figure 6:**
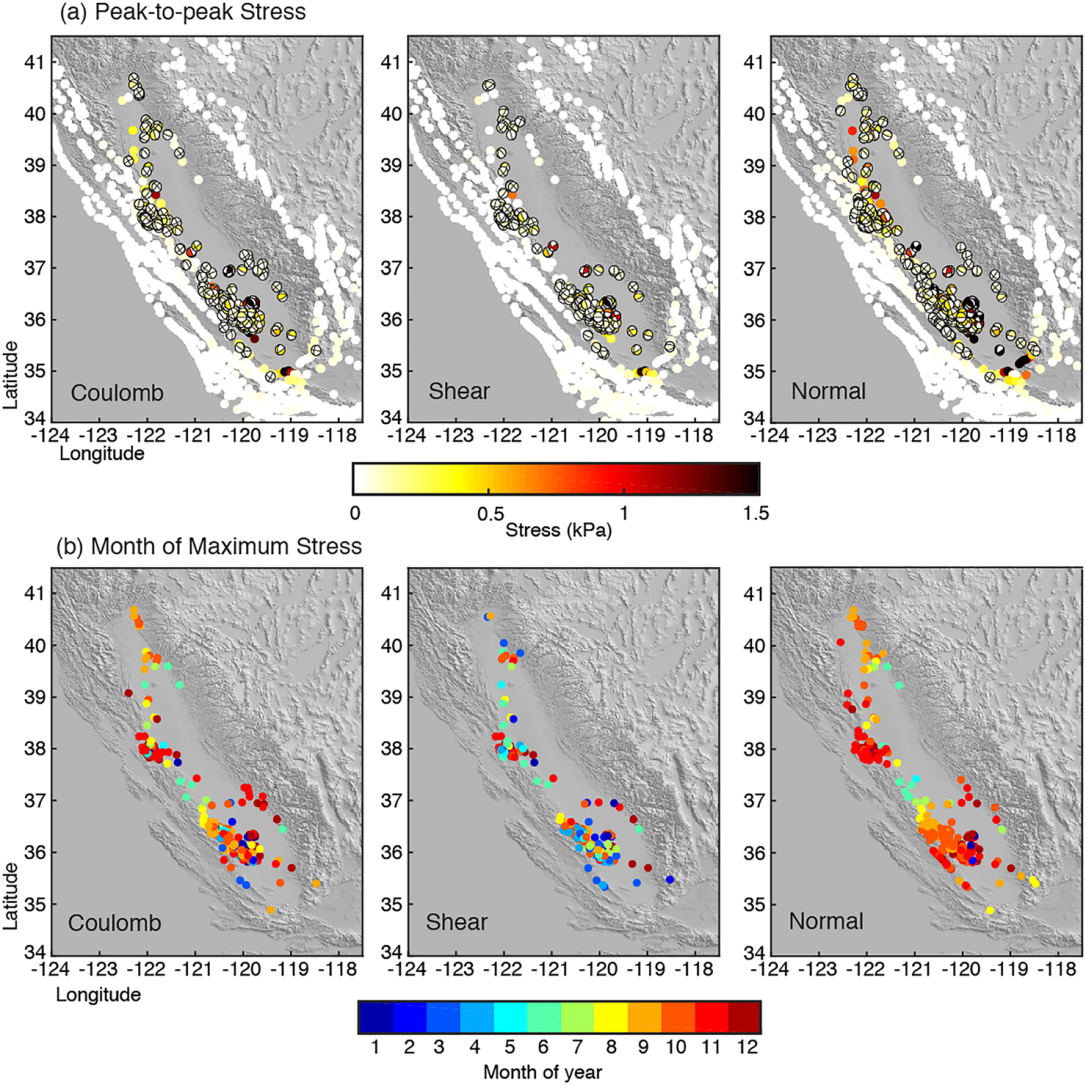
Stress change over a year due to seasonal changes in groundwater load. (a) Peak‐to‐peak Coulomb, shear, and normal stress change calculated on focal mechanism fault planes. (b) Month in which peak Coulomb, shear, and normal stress occur.

We calculate “peak‐to‐peak” stress change on focal mechanism fault planes and fault planes from the UCERF3 model by subtracting the minimum stress change from the maximum stress change experienced at each location (Figure 6a). The peak‐to‐peak Coulomb stress change across all faults in California ranges from less than 1 Pa to 2.5 kPa, with ~52% of focal mechanisms experiencing fairly insignificant peak‐to‐peak Coulomb stress change of less than 10 Pa. Peak‐to peak normal stress change ranges from ~3.1 kPa to less than 1 Pa. Peak‐to‐peak shear stress ranges from 1.6 kPa to less than 1 Pa and is overall smaller than annual normal and Coulomb stress change. As with total stress change during the drought, the largest peak‐to‐peak stress change occurs along faults near the edge of the Valley.

Figure 6b shows when peak Coulomb, shear, and normal stress occurs. Peak normal stress occurs dominantly in the fall and early winter, while peak shear stress is more variable, occurring in spring and fall. Peak Coulomb stress mostly occurs in the fall but also in the spring on faults farther north and in the late winter/early spring along parts of the San Andreas. Additionally, we find ~2‐ to 3‐month lags in the phase of peak Coulomb and normal stress change between neighboring fault segments along the Hayward Fault‐north creeping section (latitude 36.5–37.5) and between the north‐central creeping section (latitude 35.8–36.5). The San Andreas fault south of ~35.5° latitude is ~4–5 months out of phase with the rest of the fault, which might be due in part to its proximity to the irrigation‐controlled groundwater recharge cycle in the southern part of the Valley that is ~3 months out of phase with the rest of the Valley. Kreemer and Zaliapin (2018) found a similar ~4‐month phase lag for peak Coulomb stress change between the northern and southern San Andreas fault, although that study was based on seasonal horizontal strain.

### 3.4 Correlating Seasonal Stress Change With Earthquake Count and Moment Release

Seasonal groundwater storage changes impart small seasonal stress perturbations on the shallow crust. It has already been suggested that earthquake nucleation in California contains a seasonal component (e.g. Christiansen et al., 2007; Amos et al., 2014) that is likely driven by hydrospheric load changes (e.g. Amos et al., 2014; Johnson et al., 2017a, 2017b). Here we are interested in whether we see a similar correlation with groundwater volume change in the Central Valley. If these low‐amplitude periodic stresses modulate earthquake nucleation, we should see fluctuations in the seismicity rate that are coincident in time with variations of our calculated Coulomb stress change. To test this hypothesis, we randomly generate 500 synthetic earthquake catalogs that consider a seismicity rate model in which the stressing rate is constant in time (i.e., the only source of stress is steady tectonic loading) (Dieterich, 1994) and covers the same timespan as our observed catalog (January 2006 to December 2014). If seasonal groundwater volume change does help modulate seismicity, we should see a statistically significant increase in the number of events as compared to the background seismicity rate (i.e., the synthetic catalog) as Coulomb stress increases. Although we look at Coulomb, shear, and normal stress, Coulomb stress is the most important for earthquake nucleation.

**Figure 7:**
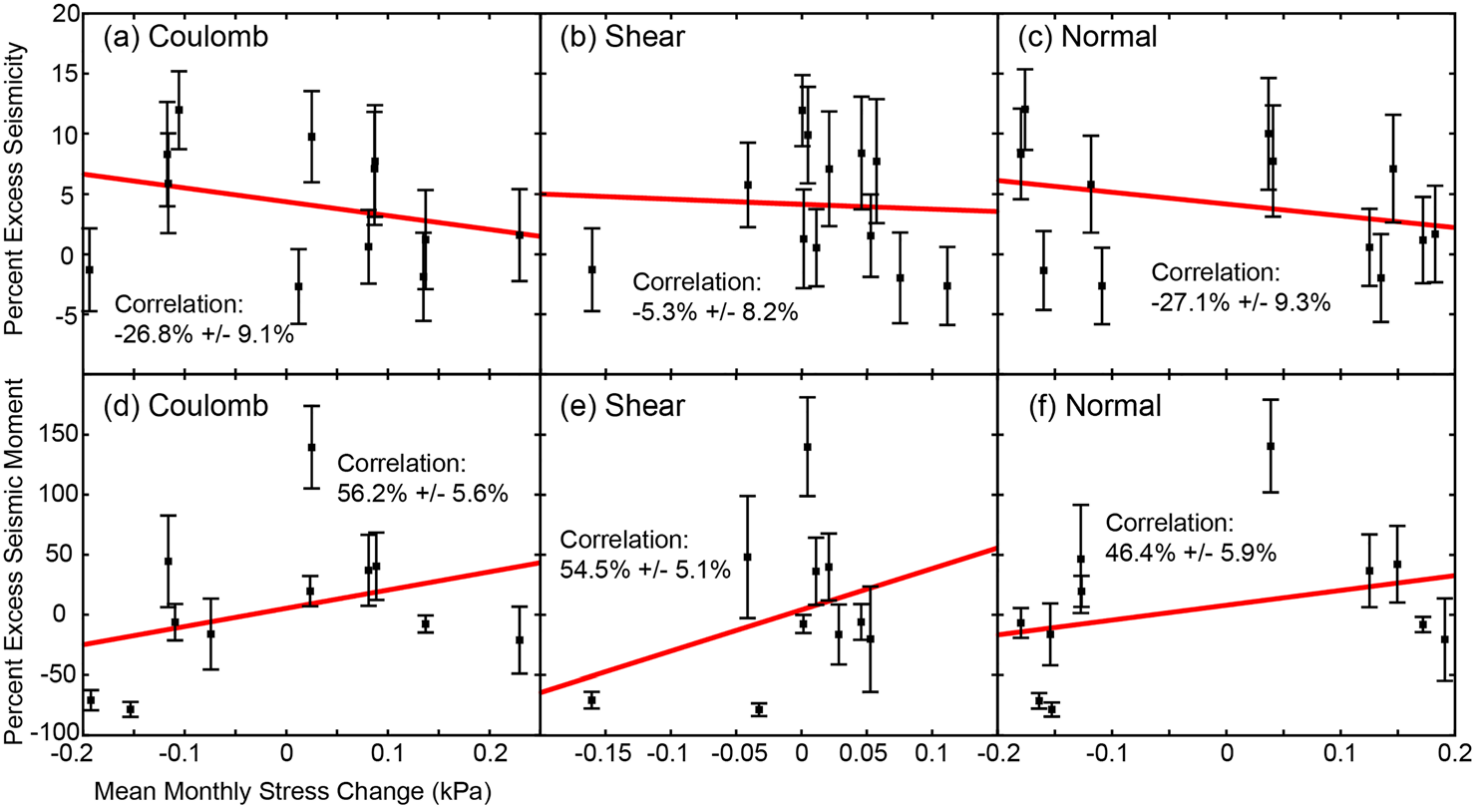
Correlation between mean monthly Coulomb (a, d), shear (b, e), and normal (c, f) stress found using the seasonal stress change values and percent excess seismicity (a–c) and percent excess seismic moment (d–f) in each month. Error bars show a 2σ error in monthly percent excess seismicity and seismic moment.

We correlate percent excess seismicity with average Coulomb, shear, and normal stress in each month. We acknowledge that our ensembles of excess seismicity include significant scattering, which is expected given the small amplitude of the hydrospheric stress changes and complexities of the seismotectonics in California. To robustly estimate the correlation, we employ the weighted Pearson correlation coefficients (Pozzi et al., 2012), in which the weight matrix is iteratively updated (Holland & Welsch, 1977) to reduce the effects of outliers on the correlation coefficient. We find negative correlations between Coulomb (−26.8% ± 9.1%), shear (−5.3 ± 8.2), and normal (−27.1 ± 9.3) stress change and percent excess seismicity (Figures 7a–7c). Given that many faults experience near‐zero stress change and that more earthquakes in our catalogue occur in spring (Figure S2) when seasonal stress is at a minimum along many faults, the negative correlations are not surprising.

We also correlate mean Coulomb, shear, and normal stress with percent excess seismic moment release. Seismic moment release can help explain the relative proportion of small and large earthquakes. Because our synthetic catalog samples from a probability distribution, the chances of randomly selecting earthquakes greater than magnitude 5 is low; however, our observed catalog contains earthquakes up to magnitude 6.2 (August, 2014 Napa earthquake) (Figure S2). To compare the moment release in these two catalogs, we remove earthquakes greater than magnitude 5 from both catalogs. The excess seismic moment correlated with mean Coulomb, normal, and shear stress change is shown in Figures 7d–7f. We find positive correlations between mean Coulomb, shear, and normal stress change and percent excess seismic moment. The most significant correlation is between mean Coulomb stress change and excess seismic moment with a percent correlation of 56.2% ± 5.6%. Positive correlations between seismic moment release and higher‐than‐average stress show that although fewer earthquakes occur in the fall when seasonal groundwater‐loading induced stress is maximum along many faults, the earthquakes that do occur are larger.

## 4 Discussion and Conclusions

### 4.1 Importance of High‐Resolution Groundwater Volume Change Estimates

This study demonstrates the importance of utilizing poroelastic as well as elastic deformation when quantifying TWS changes and associated loading stresses in regions with large aquifer systems. Our results indicate that elastic deformation due to groundwater storage change is mostly absent in GPS vertical displacement time series used for TWS estimates, although poroelastic deformation is visible within the Valley. Thus, estimates of seasonal and long‐term TWS change and associated crustal stress perturbations may be underestimated. Accurate modeling of vertical deformation due to elastic loading effects also has implications for studies that use GPS data and wish to remove the groundwater loading signal in order to isolate tectonic signals. If the elastic loading model is inaccurate, then the corresponding correction will also be inaccurate. Our tests using different smoothing kernels show that smoothing the groundwater volume change data using the Gaussian filter of 50‐km width underestimates the predicted vertical signal at the center of the load and slightly overestimates the signal from the rough edge of the Valley to 100 km outside of the Valley (~50–150 km from the center of the load). The 300‐km width Gaussian filtered data set underestimates the predicted signal across the entire area, likely because the filter radius is larger than the width of the Valley and just slightly smaller than the total width of our study area (~200 km).

Spatial smoothing of the groundwater loading signal leads to differences in calculated stress changes as well. Within a certain distance from the center of a load, a reduction in mass causes reduced overburden pressure and leads to unclamping, making a fault more likely to rupture. Thus, normal stress is considered the dominant control on loading‐induced stress under the source of loading. Outside of this zone of tension, though, shear stress plays a more significant role. When we apply Gaussian smoothing filters to the data, we extend the width of the load area, thus extending the zone with significant tensile normal stress and near‐zero shear stress. This is why when looking at the unfiltered data, most faults show a smaller shear stress change than the unfiltered data set. The 300‐km Gaussian filtered data set significantly underestimates calculated stress change. Therefore, we believe the currently achievable spatial resolution of GRACE is not sufficient for hydrospheric loading induced stress calculations. Improving the spatial resolution of GRACE using GPS or InSAR will improve these types of crustal stress estimates.

### 4.2 Seasonal Stress Variations and Implications

We find that groundwater loading imparts variable periodic stress on faults depending on the orientation of the fault and proximity to the source of loading. Moreover, the periodic stress variation due to water storage change is likely to produce a variable influence on modulation of earthquakes because of the complex interactions between neighboring faults, along‐strike variability in frictional properties, the inherent randomness of earthquake occurrence, and stress shadowing from large earthquakes, to name a few examples. Additionally, because we only look at stress change from groundwater volume change in the Central Valley, we can only explain a portion of the harmonic stress perturbations that influence these faults. Incorporating changes in other terrestrial water storages such as snowcap, soil moisture, and surface water as well as thermoelastic stresses, atmospheric pressure and groundwater volume change from other aquifer systems in the state that are not considered (e.g., the Coastal Basin and Pacific Northwest aquifers), will likely increase the calculated seasonal stressing amplitude, depending on the period and phase of their loading cycle. According to Johnson et al. (2017b), though, hydrologic‐loading stresses in California are the dominant nontectonic source of stress and temperature, atmospheric pressure, and tidal loading play only minor roles. Additionally, Groundwater storage change is driven by both natural cycles and human consumption, unlike other TWS components, which are driven primarily by natural cycles. Thus, these results indicate that anthropogenic water use is likely adding to seasonal stress, particularly along faults close to the Valley edge.

Shear and normal stress change are out of phase along much of the fault. Normal stress tends to peak in the late fall and early winter, while shear stress peaks dominantly in both the late fall and the spring. Because Coulomb stress change is the addition of shear and normal stress multiplied by a coefficient of friction, the timing of peak Coulomb stress is controlled by the timing and amplitudes of shear and normal stress. This makes the timing of peak Coulomb stress more variable, although it dominantly follows peak normal stress.

### 4.3 Correlating Periodic Stress Change With Excess Seismicity and Seismic Moment

Laboratory experiments and modeling studies demonstrate that if the amplitude of harmonic stress perturbations is sufficient (of the same order of magnitude as the tectonic stressing rate) and the period of stressing is longer than the nucleation time, then harmonic stresses can influence the timing of earthquakes and the balance between small and large earthquakes (Ader et al., 2014; Beeler & Lockner, 2003). These studies predict that if the ratio of $\frac{2\pi }{T}\mathrm{Amp}$, where *T* is the period and *Amp* is the amplitude of stress, to the secular loading rate is greater than 1, then there is likely to be a correlation between the annual stress and earthquake occurrence (Lockner & Beeler, 1999; Beeler & Lockner, 2003; Heki, 2003). According to Smith and Sandwell (2003), the Coulomb stressing rate along faults in California ranges from ~5 to 125 kPa/year. For most faults, stress from groundwater unloading is much too small to impact earthquake modulation and any correlation between earthquake count and stress will not be able to be differentiated from random earthquake occurrence. Some faults close to the Valley, though, receive high annual Coulomb stress change with an amplitude of ~1.3 kPa and low tectonic stressing rates of ~5‐40 kPa/year. This results in a periodic to secular stress ratio of ~0.2–1.6. Thus, there is a stronger likelihood that faults near the Valley are modulated by groundwater loading but likely are not modulated by groundwater alone. Other studies have noted that if the fault is critically stressed, small harmonic perturbations can push a fault to failure (Tanaka, 2012). Thus, although these stress perturbations from groundwater unloading and hydrologic stress fluctuations in general are small compared to the background tectonic stress, periods of higher‐than‐average stress might help to push a fault to rupture if it is already near failure or an earthquake might be larger than normal if it occurs during peak harmonic stressing (Ader et al., 2014; Kreemer & Zaliapin, 2018). For these reasons, harmonic stress perturbations should be included in earthquake hazard prediction and probability assessments.

We also recognize that there are biases introduced in both the real focal mechanism catalog and our synthetic catalog. The minimum magnitude of completeness determination, as well as declustering, introduce biases in the real catalog. However, because this catalog is previously published (Johnson et al., 2017a), we feel it gives a good representation of seismicity in California and allows for easy comparison between the results of this study and others. The spatial and temporal scale of our study is a limiting factor and might also introduce biases. Our synthetic catalog is also likely biased by our choices of *b* value and productivity rate, and by assuming those values are spatially and temporally homogeneous. We show that seasonal stressing amplitudes and total stress change during the 2007–2010 drought are variable along faults in California and also recognize the inhomogeneity in the state of stress and frictional properties of faults considered in this study. Therefore, in the future, it may be necessary to look at nontectonic stress perturbations and correlation with seismicity through a local lens rather than a regional lens as other studies have done and as we have done in this study.

Despite the limitations expressed, our results provide important indications that groundwater volume change and long‐term loss in the Central Valley aquifer system does modify crustal stress along faults near the Valley edge and must be more accurately measured in order to improve TWS change and hydrological loading stress estimates. This study is the first independent estimate of groundwater‐load induced stress changes and is important for how we consider anthropogenic hazards associated with pumping activities in the Central Valley. Ultimately, this helps us better understand the interactions between the hydrosphere, human activity, and the solid Earth.

## Supporting information



Supporting Information S1

Table S1

Table S2

Table S3

Table S4
