# Supplementary material for: Seasonal and Long‐Term Groundwater Unloading in the Central Valley Modifies Crustal Stress
Source: J Geophys Res Solid Earth. 2020 Jan 20;125(1):e2019JB018490. doi: 10.1029/2019JB018490 (PMC7607378; doi:10.1029/2019JB018490)
Supplement: Supplementary file 1 — Supporting Information S1 [file JGRB-125-e2019JB018490-s005.docx]

*Journal of Geophysical Research: Solid Earth*

Supporting Information for

**Seasonal and Long-term Groundwater Unloading in the Central Valley Modifies Crustal Stress**

G. Carlson,^1*^ M. Shirzaei^1^, S. Werth^1,2^, G. Zhai^1,3^, C. Ojha^1^

^1^ School of Earth and Space Exploration, Arizona State University, Tempe, AZ, USA

^2^ School of Geographical Sciences & Urban Planning, Arizona State University, Tempe, AZ, USA.

^3^ Department of Earth and Planetary Science, University of California, Berkeley, Berkeley, CA, USA

**Contents of this file:**

Text S1

Figures S1 to S5

**Additional Supporting Information (Files uploaded separately):**

Captions for Tables S1 to S4

**Introduction**

Contained in this document is:

1. A correlation between seasonal stress change and excess seismicity for events that have peak-to-peak stress greater than 0.05 kPa or less than -0.05 kPa. In this section is a histogram showing when all earthquakes happen in our real catalogue and the spread of associated stress change in the month in which each occurrs.
2. Synthetic versus real seismic catalogue.
3. UCERF3 fault strike, dip, and rake.
4. 5 data files containing the inputs into STATIC1D for the four different models (unfiltered rate, 50-km Gaussian filtered rate, 300 km Gaussian filtered rate, monthly inputs) and the variables for the seasonal stress correlation tests.

**Text S1. Correlating the stress change with earthquake count for events significantly impacted by groundwater-load induced stress changes**

We calculate the correlation between excess seismicity and stress change using only events that have a stress change greater than 0.05 kPa or less than -0.05 kPa. This threshold is set based on the lowest estimates of long-term coulomb stressing rates in our study area (Smith and Sandwell, 2003). We do not expect stress changes smaller than this to have any effect on earthquake timing. Even sections of the fault that experience seasonal fluctuations of ~1-0.05 kPa, are unlikely to exert a strong control on earthquake timing because the static Coulomb stress accumulation rate is at the lowest estimate ~5 kPa/yr (Smith and Sandwell, 2003), which is much larger than the groundwater-load induced seasonal stress fluctuations we calculate (Vidale et al., 1998; Lockner & Beeler, 1999; Beeler & Lockner, 2003). We include this in the supplement instead of in the main text because less than 10% of mechanisms show stress changes in this range (Figure S4), thus we are not confident that the correlation is legitimate.

Groundwater-load change induced stress change, independent from TWS change- induced stress change, mildly correlates with seismicity fluctuations if we consider only those earthquakes that have stress change greater than 0.05 kPa or less than -0.05 kPa (S3). If we consider all earthquakes in the catalogue, we find negative correlations between mean Coulomb and normal stress change and excess seismicity and a weak positive correlation between shear stress change and excess seismicity (Figure 7). This is likely because the annual stressing amplitude is much smaller than static Coulomb stress accumulation, thus tectonic loading is the dominant control on earthquake nucleation.


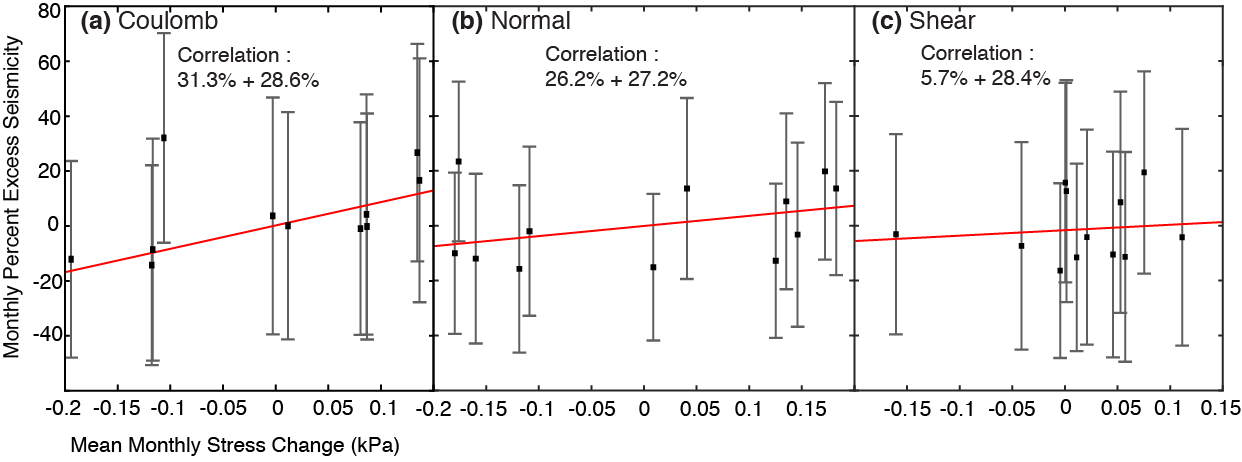


**Figure S1.** Correlations between **(a)** Coulomb, **(b)** normal, and **(c)** shear stress change for events that experience peak-to-peak stress greater than 0.05 kPa.


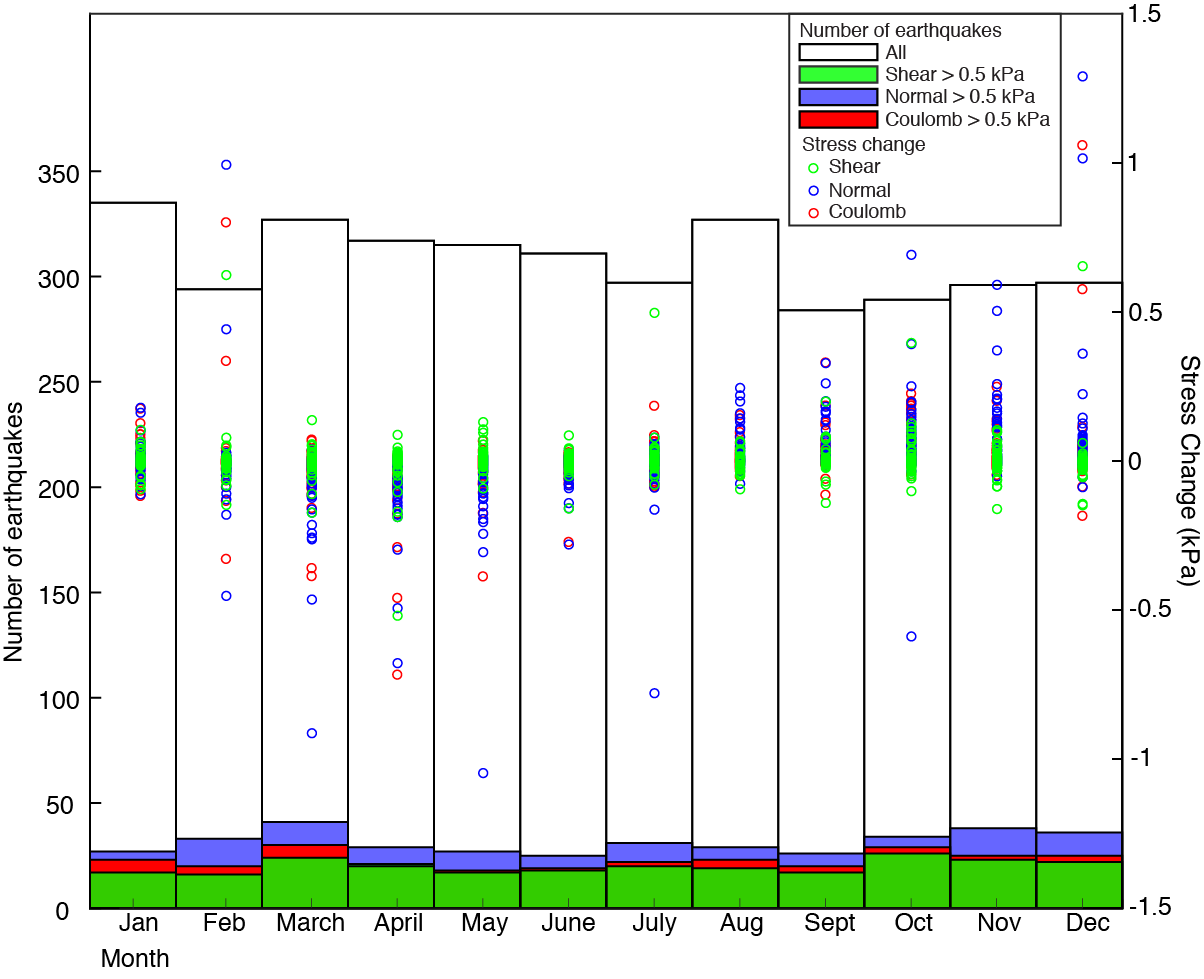


**Figure S2.** Histogram of earthquakes that occur in each month (white bars) as well as the number of events in each month that experience peak-to-peak Coulomb stress > 0.05 kPa (red), shear stress > 0.05 kPa (green) and normal stress > 0.05 kPa (blue). The histogram corresponds to the left y-axis. This is overlain by Coulomb (red), shear (green), and normal (blue) dots, showing stress calculated on focal mechanism fault planes in the month that each earthquake occurs. The dots correspond to the right y-axis. This plot shows that maximum stress is dominantly in the fall, when more earthquakes occur that feel larger harmonic stress perturbations from groundwater loading (shown by the colored histogram values). However, when stress is at a minimum in March, there also seems to be a larger number of earthquakes.


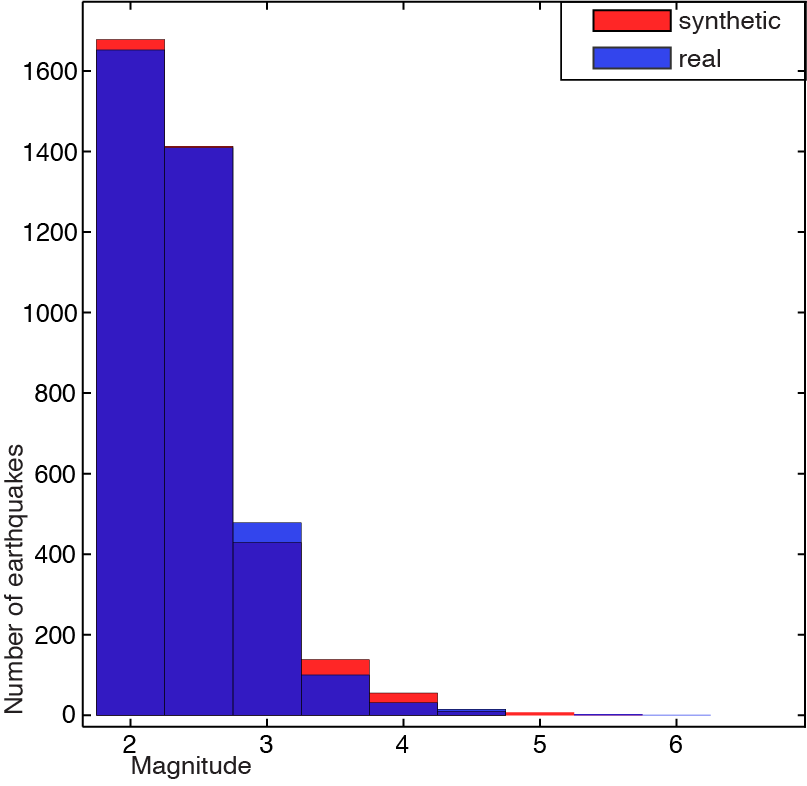


**Figure S3**. Magnitude-frequency histogram of earthquakes in the real and synthetic catalogues.


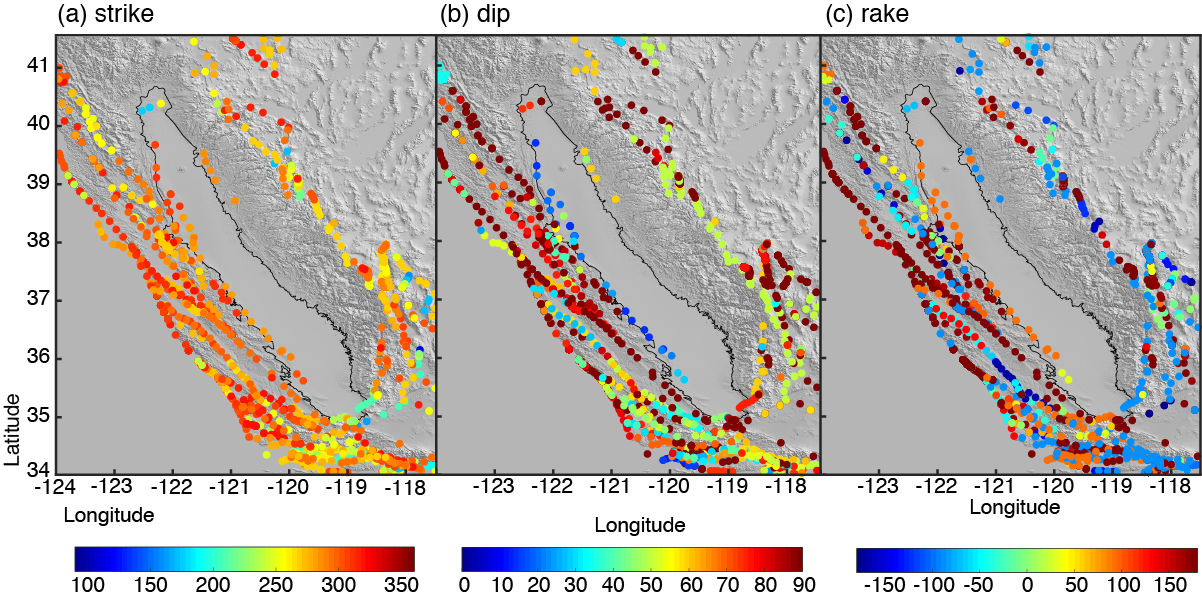


**Figure S4.** Strike, dip, and rake of the UCERF model. Stress change is calculated along these fault planes and a depth of 8km.


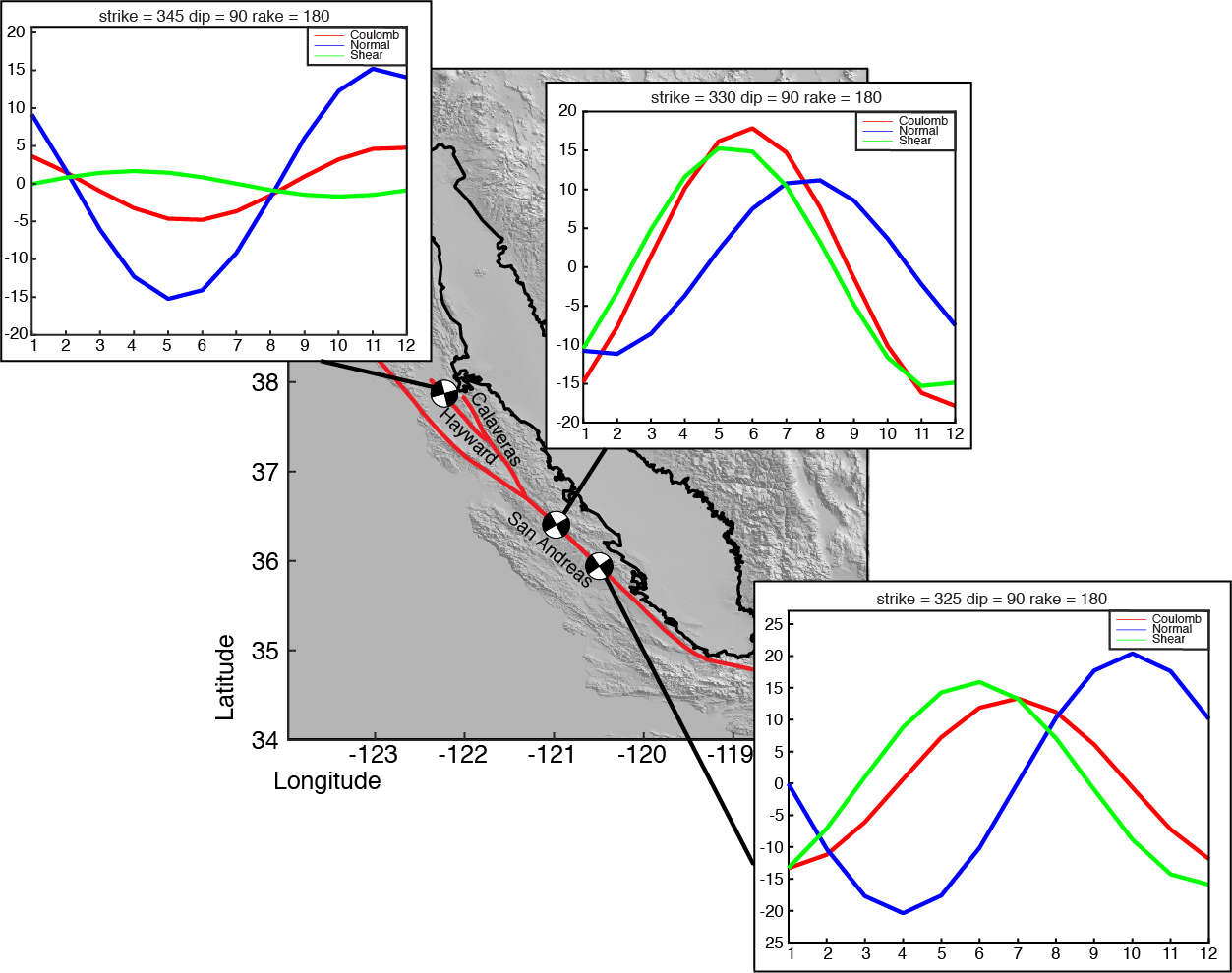


**Figure S5**. Fluctuations of Coulomb (red), shear (green), and normal (blue) along the Hayward fault in the north and San Andreas fault in the south. All stress change values are given in Pa. We can see that stress peaks vary along the Hayward fault in the North to the end of the creeping section in the south. Although the strike of the fault is the same, the angle from the groundwater load changes. Additionally, the groundwater load changes in the south of the Valley are dominated by irrigation activity, which lags the natural peak groundwater recharge in the north by ~3 months. Thus, variability in the timing of maximum stress is controlled by the spatial variability in the amplitude of volume change and the time at which the peak occurs in addition to the strike and dip of the fault.

**Table S1.** Input for modified version of STATIC1D using our unfiltered total groundwater loss.

**Table S2.** Input for modified version of STATIC1D using our unfiltered total groundwater loss smoothed using a 50 km Gaussian filter.

**Table S3.** Input for modified version of STATIC1D using our unfiltered total groundwater loss smoothed using a 300 km Gaussian filter.

**Table S4.** Input for modified version of STATIC1D monthly volume of groundwater loss found using phase and amplitude information.
